# Polyunsaturated fatty acids as a potential preventive and therapeutic intervention for metabolic dysfunction–associated steatotic liver disease and its progression to hepatocellular carcinoma

**DOI:** 10.3389/fnut.2026.1767917

**Published:** 2026-02-11

**Authors:** Thomai Kouti, Panayiota Christodoulou, Stephanos Christodoulides, Foula Protopapa, Charalambos Michaeloudes, Paraskevi A. Farazi

**Affiliations:** 1 School of Medicine, European University Cyprus, Egkomi, Nicosia, Cyprus; 2 School of Sciences, European University Cyprus, Egkomi, Nicosia, Cyprus

**Keywords:** HCC, MASH, MASLD, omega 3 (n-3) polyunsaturated fatty acids, omega-6 PUFAs, PUFA

## Abstract

Metabolic dysfunction–associated steatotic liver disease (MASLD) is currently the leading cause of chronic liver disease worldwide and a major cause of hepatocellular carcinoma (HCC), a cancer with poor prognosis. Considering the immense public health impact of MASLD and MASLD-HCC, preventive and more effective management strategies for these diseases are urgently needed. Polyunsaturated fatty acids (PUFAs) appear to improve liver health through modulation of lipid metabolism, inflammation and oxidative stress and therefore could influence MASLD and MASLD-HCC progression. To this end, this review discusses the role of PUFAs, more specifically n-3 and n-6, in MASLD and MASLD-HCC, by critically reviewing evidence from human clinical and observational studies, and experimental models. Human observational and clinical trial studies collectively suggest a beneficial effect of PUFAs in the prevention of MASLD and MASLD-HCC. Evidence in animal models indicate that n-3 PUFA supplementation suppresses the development of MASLD by preventing liver steatosis, inflammation, and fibrosis. These effects are mediated through a shift in lipid metabolism from lipogenesis toward lipolysis and fatty acid oxidation, inhibition of key inflammatory pathways and antioxidant effects. There is evidence from a small number of animal model studies showing a reduction in PUFA levels during MASLD progression to HCC, and a protective effect of n-3 PUFA supplementation against liver tumorigenesis. However, the evidence on the molecular mechanisms mediating this effect is very sparse. The evidence reported in this review suggests consideration of PUFAs, and particularly n-3 PUFAs, as potential preventive modalities for MASLD-HCC and for control of established MASLD-HCC in combination with existing therapies, albeit in a microenvironment context-dependent manner. Finally, the review highlights key gaps in the literature and suggests potential research opportunities to delineate the role of PUFAs in MASLD-HCC.

## Introduction

Metabolic dysfunction–associated steatotic liver disease (MASLD), previously known as non-alcoholic fatty liver disease (NAFLD), represents the hepatic manifestation of metabolic syndrome and is now the leading cause of chronic liver disease worldwide (1). 38% of all adults worldwide suffer from MASLD and by 2040, the prevalence is expected to reach 55% (2). A subset of patients progress to metabolic dysfunction–associated steatohepatitis (MASH), characterized by hepatocellular injury, inflammation, and varying degrees of fibrosis. Over time, MASH can evolve into advanced fibrosis, cirrhosis, and eventually hepatocellular carcinoma (HCC), even in the absence of cirrhosis, highlighting the disease’s oncogenic potential and its rising global burden (3, 4). HCC is the leading type of primary liver cancer and a significant contributor to cancer-related deaths globally. In addition to MASLD, other chronic liver conditions, such as viral hepatitis and alcoholic liver disease are associated with HCC. However, in recent years, an epidemiologic shift has occurred for HCC with an increasing number of HCC cases now being associated with MASLD (5).

There is currently no available therapy specifically targeting MASLD-HCC patients, who are treated for liver cancer, based on tumor stage, by surgery, non-specific chemotherapy, or immunotherapy. Patients, therefore, experience significant side effects from the chemotherapy, whilst a proportion of patients may respond poorly to immunotherapy (6). Therefore, therapies targeting metabolic dysfunction may enable a more personalized and targeted treatment approach for MASLD-HCC patients.

Given the emerging evidence for the role of PUFAs in modulating processes of metabolic dysfunction including lipid metabolism, inflammation, and oxidative stress, it is reasonable to consider their use in the prevention and control of MASLD and MASLD-HCC. Epidemiologic and animal model data support the role of PUFAs, particularly n-3 PUFAs, in the prevention of MASLD. However, although epidemiological evidence supports a potential protective effect of PUFAs against the development of MASLD-HCC, there is a lack of data from experimental models and a poor understanding of the mechanisms involved. To paint a clearer picture on this topic this review will: (1) Synthesize human evidence for PUFAs in preventing and managing MASLD and MASLD-HCC through a comprehensive review of the existing epidemiological, clinical and experimental data; (2) Detail the established mechanisms by which PUFAs improve MASLD and MASLD-HCC; (3) Critically examine the conflicted and emerging evidence for PUFAs in MASLD-HCC, focusing on the unique MASLD tumour microenvironment; and (4) Outline priority research directions to trigger progress in filling the gaps in this area. This review is the first to address the role of PUFAs beyond MASLD and dive into their role in the progression to MASLD-HCC with the aim to highlight controversies and research gaps in this field. Considering the poor survival of MASLD-HCC patients, understanding how PUFAs may impact MASLD-HCC development and the mechanisms associated with their tumor effects are of utmost importance.

## Pathogenesis of MASLD/MASLD-HCC

MASLD is the most common cause of chronic liver disease and has a higher prevalence in people with obesity, type 2 diabetes, and metabolic syndrome. It is characterized by excess lipid accumulation within hepatocytes in the context of metabolic dysfunction, typically linked to insulin resistance. It involves steatosis affecting more than 5% of the liver parenchyma, reflecting an imbalance between lipid acquisition and disposal within the liver (7). In addition, the prevalence of MASLD is associated with genetic polymorphisms in genes such as *PNPLA3*, *TM6SF2*, *MBOAT7*, and *HSD17B13*, which make individuals susceptible to fat accumulation, inflammation, and fibrosis (8). The prevalence of the disease also varies geographically, with higher prevalence reported in South America and the Middle East, and lower prevalence in Africa. These prevalence patterns reflect differences in lifestyle, diet and genetic susceptibility. MASLD is a leading indication for liver transplantation and a rapidly growing risk factor of HCC, even in the absence of cirrhosis (9). MASLD is the recently adopted term that replaces non-alcoholic fatty liver disease (NAFLD) to better reflect its metabolic origins. Traditionally, NAFLD referred to hepatic fat accumulation not caused by excessive alcohol intake and encompassed two stages: non-alcoholic fatty liver (NAFL), characterized by simple steatosis without significant inflammation, and non-alcoholic steatohepatitis (NASH), where steatosis is accompanied by hepatocellular injury, inflammation, and varying degrees of fibrosis (10). In 2023, international liver societies introduced the term MASLD, defining it as hepatic steatosis in individuals with at least one cardiometabolic risk factor such as obesity, type 2 diabetes, dyslipidemia, or hypertension. The progressive form, metabolic dysfunction–associated steatohepatitis (MASH), corresponds to NASH in the older terminology and represents the stage most likely to progress to cirrhosis and HCC (11). The metabolic origins of MASLD render it different from other types of chronic liver diseases, which requires different considerations in prevention and treatment of MASLD and MASLD-HCC.

**Figure 1:**
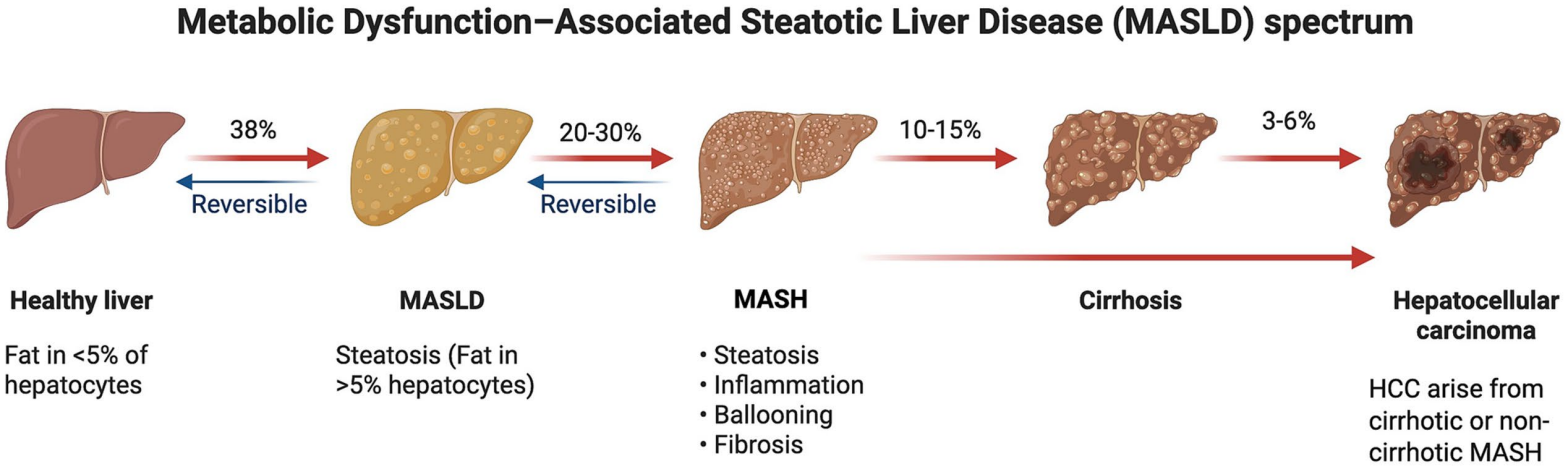
Metabolic dysfunction–associated steatotic liver disease (MASLD) spectrum and modulatory effects of polyunsaturated fatty acids (PUFAs). Schematic representation of the progression of metabolic dysfunction–associated steatotic liver disease (MASLD), beginning with a healthy liver and advancing through MASLD, metabolic dysfunction–associated steatohepatitis (MASH), cirrhosis, and ultimately hepatocellular carcinoma (HCC). Approximate progression rates between stages are shown based on current epidemiological evidence. Created in https://BioRender.com.

Progression of MASLD to metabolic dysfunction–associated steatohepatitis (MASH) occurs in about 20–30% of MASLD patients. Individuals with MASH are at increased risk of fibrosis, cirrhosis, and hepatocellular carcinoma (HCC), and among those who develop MASH, approximately 10–15% will progress to cirrhosis (12). Overall, an estimated 3–6% of patients with MASH will eventually develop MASH-associated hepatocellular carcinoma (HCC) (13). Notably, among individuals who develop MASH-related HCC, a substantial proportion arises in the absence of preceding cirrhosis (14, 15) (Figure 1). The histological changes of MASLD follow a pattern of changes very similar to that of the alcohol induced hepatic injury and range from simple fat accumulation to inflammation, fibrosis and cirrhosis. The “multiple hit hypothesis” suggests that the development of MASLD stems from metabolic, inflammatory, and genetic factors. Insulin resistance leads to an increase in adipose tissue lipolysis, and then an influx of free fatty acids into the liver, promoting hepatic steatosis. When free fatty acids, diacylglycerols and ceramides accumulate in the liver, the mitochondrial *β*-oxidation of these molecules is impaired, resulting in the overproduction of reactive oxygen species (ROS) and oxidative stress. This causes lipid peroxidation, cell membrane damage and hepatocyte apoptosis or necrosis, leading to the release of damage-associated molecular patterns (DAMPs) (16). The gut-liver axis plays a key role in MASLD pathogenesis, as the intestinal dysbiosis increases the permeability of the gut, allowing bacterial endotoxins to reach the liver through the portal vein. DAMPs and endotoxins activate receptors, such as toll-like receptors (TLRs), in Kupffer cells, which trigger inflammatory mechanisms, including transcription factor NF-κB, mitogen associated protein kinases (MAPKs) and the NOD-like receptor protein 3 (NLRP3) inflammasome (17). The resulting inflammatory response is associated with the release of cytokines such as TNF-*α*, IL-6, and IL-1*β*, and growth factors, including transforming growth factor (TGF)-β. These mediators promote hepatic stellate cells to transform into myofibroblasts, which in turn produce extracellular matrix components, resulting in fibrosis. As these processes progress, they disrupt the architecture and function of the liver, paving the way for cirrhosis and HCC (18).

HCC develops through a complex interplay of genetic, epigenetic, metabolic, and inflammatory alterations that transform chronically injured hepatocytes into malignant cells. In MASLD and MASH, persistent lipotoxicity and oxidative stress induce DNA damage, mitochondrial dysfunction, and impaired repair mechanisms. ROS and lipid peroxidation products such as malondialdehyde (MDA) and 4-hydroxynonenal (4-HNE) form mutagenic adducts with DNA, promoting genomic instability (19). Continuous activation of inflammatory signaling, notably NF-κB, JNK, and STAT3 pathways, drives hepatocyte proliferation, survival, and resistance to apoptosis (20). At the same time, metabolic reprogramming in pre-malignant hepatocytes supports tumorigenesis. Enhanced *de novo* lipogenesis, altered *β*-oxidation, and a shift toward aerobic glycolysis provide energy and biosynthetic precursors for rapid cell growth (21). Oncogenic pathways such as Wnt/β-catenin, PI3K/Akt/mTOR, and MAPKs become aberrantly activated, either through mutations or chronic signaling, leading to uncontrolled proliferation and angiogenesis (22). Epigenetic modifications, including DNA hypermethylation, histone acetylation, and deregulated microRNAs (e.g., miR-122, miR-21), further silence tumour suppressor genes and enhance oncogene expression (23). Collectively, these molecular alterations enable hepatocytes to escape normal growth controls and acquire malignant potential.

The HCC tumor microenvironment plays a pivotal role in promoting tumor initiation, progression, and immune escape. Chronic liver injury remodels the hepatic niche into a pro-inflammatory, fibrotic, and immunosuppressive milieu. Activated hepatic stellate cells (HSCs) and cancer-associated fibroblasts (CAFs) secrete extracellular matrix components, TGF-*β*, and vascular endothelial growth factor (VEGF), fostering fibrosis and neovascularization. This fibrotic matrix not only supports tumor cell proliferation but also provides structural scaffolding for invasion and metastasis (24). Kupffer cells and infiltrating macrophages adopt a tumor-promoting (M2-like) phenotype, releasing IL-6, TNF-*α*, and ROS, which further enhance inflammation and oncogenic signaling (25). Chronic hypoxia in the fibrotic liver upregulates hypoxia-inducible factors (HIF-1α and HIF-2α), stimulating angiogenesis and metabolic adaptation of cancer cells (26). In parallel, the TME becomes immunosuppressive, with regulatory T cells (Tregs), myeloid-derived suppressor cells (MDSCs), and exhausted cytotoxic T cells impairing effective antitumor immunity. Tumor cells exploit immune checkpoints such as PD-1/PD-L1 and CTLA-4 to evade immune surveillance (27). Altogether, these cellular and molecular interactions create a self-reinforcing loop of inflammation, fibrosis, and immune tolerance that sustains tumor growth and progression. The unique tumor and microenvironment characteristics in MASLD-HCC require special considerations for the prevention and treatment of the disease, which may be different from HCC of other etiologies.

## Current management strategies for MASLD and MASLD-HCC

**Figure 2:**
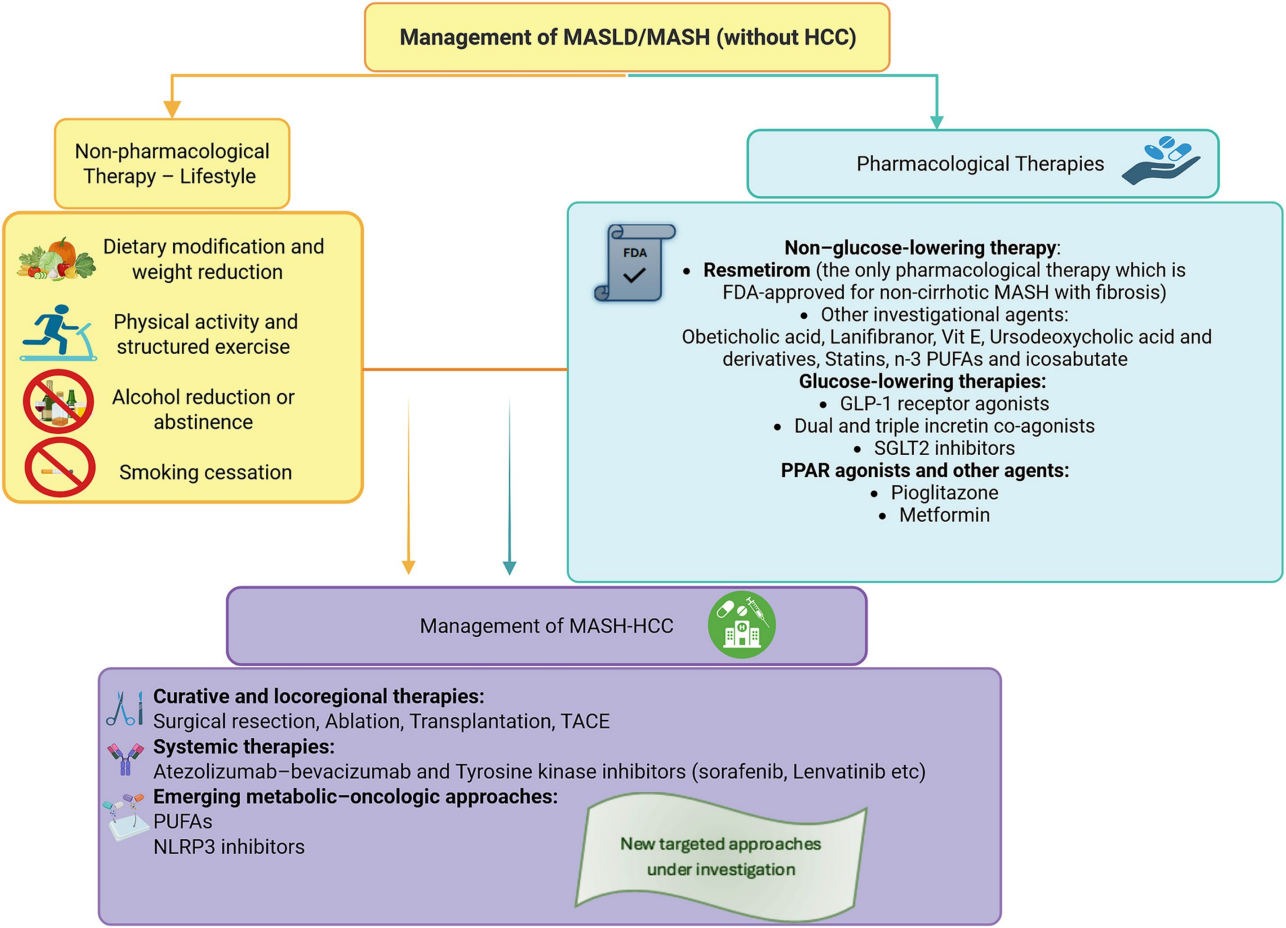
Management for MASLD/MASH with and without hepatocellular carcinoma (HCC). This figure provides an overview of current management approaches for metabolic dysfunction–associated steatotic liver disease (MASLD) and metabolic dysfunction–associated steatohepatitis (MASH), with and without hepatocellular carcinoma (HCC). It illustrates both established therapeutic options and investigational treatments that are currently being evaluated in clinical trials. Created in https://BioRender.com.

Although the selective thyroid hormone receptor-*β* (THR-β) agonist resmetirom is currently the only approved pharmacologic therapy for MASH, the management of MASLD and MASH remains predominantly non-pharmacological (16, 28). Current strategies focus on lifestyle modification, weight reduction, dietary interventions, increased physical activity, and optimization of metabolic comorbidities, which continue to represent the cornerstone of disease prevention and treatment (Figure 2) (7). Particular attention is directed toward the management of key metabolic comorbidities such as obesity, insulin resistance, and dyslipidemia, which constitute major therapeutic targets in slowing disease progression and reducing overall cardiometabolic risk (16, 29, 30). As far as MASLD-HCC is concerned, there are no specific treatments for the disease. Instead, currently available treatments for HCCs of other aetiologies are also used for the management of MASLD-HCC.

### Management of MASLD/MASH

#### Non-pharmacological treatments in MASLD/MASH

The primary objective of disease management is to achieve a clinically meaningful improvement in patient outcomes. In liver disease, these outcomes typically include the prevention of cirrhosis decompensation, preservation of liver function, reduction in the risk of HCC, and avoidance of liver transplantation (31). Within this framework, non-pharmacological interventions represent a cornerstone in improving clinical outcomes and slowing disease progression, encompassing dietary modification and weight reduction, regular physical activity and structured exercise programs, as well as reduction or abstinence from alcohol consumption and smoking cessation (32).

Weight loss remains the most effective non-pharmacological intervention for managing MASLD, with consistent evidence supporting a dose-dependent reduction in liver fat content, steatohepatitis, and fibrosis (32). According to the EASL–EASD–EASO Clinical Practice Guidelines, targeted weight reduction is a cornerstone in MASLD management, with specific goals tailored to body mass index (BMI) and clinical context. In overweight or obese individuals, a ≥ 5% weight loss reduces hepatic steatosis, while a 7–10% loss is typically needed to improve MASH and hepatic inflammation, and ≥10% to reverse fibrosis (33). For patients with class II or III obesity, incretin-based pharmacotherapy or bariatric surgery may be considered, whereas even modest weight loss (3–5%) in individuals with normal BMI can improve hepatic steatosis (7).

Numerous clinical trials have demonstrated that weight loss achieved through caloric restriction leads to improvements in liver enzymes, hepatic steatosis, fibrosis, and MASH progression (33–36). Furthermore, weight reduction contributes to better glycemic control, lipid profiles, and blood pressure regulation, while also lowering the risk of cardiovascular disease and other metabolic complications (37). However, longitudinal studies indicate that maximal weight loss tends to occur at around 6 months, followed by partial weight regain, with a net average weight loss of approximately 5%, and an associated partial reaccumulation of hepatic fat and stiffness by 12–24 months (7, 38).

Various nutritional approaches have been investigated in this context, including hypocaloric low-carbohydrate and low-fat diets, Mediterranean-style eating patterns, very low-carbohydrate ketogenic diets, and intermittent fasting strategies such as time-restricted eating (TRE). Within this spectrum, the Mediterranean diet has drawn considerable attention, as it emphasizes minimizing the intake of processed and ultra-processed foods, such as processed meats and sugar-sweetened beverages, while increasing the consumption of unprocessed or minimally processed foods. The Mediterranean diet, rich in fruits, vegetables, whole grains, fish, and olive oil, provides a high content of monounsaturated and n-3 PUFAs that can reduce hepatic fat accumulation, enhance insulin sensitivity, and improve steatosis even in the absence of significant weight loss. Additionally, it may modulate the gut microbiota, thereby attenuating hepatic inflammation and supporting metabolic function (39). The Mediterranean diet has demonstrated additional benefits in reducing hepatic lipid accumulation and enhancing cardiometabolic health, and it may offer greater long-term adherence compared to other dietary approaches (40–42). Beyond the Mediterranean pattern, more restrictive approaches such as very low-carbohydrate ketogenic diets have also been examined. Evidence on the efficacy and safety of very low-carbohydrate ketogenic diets (<20–50 g/day) in MASLD is currently limited, and potential cardiovascular and renal risks warrant caution (43, 44). Another dietary intervention that has gained interest is intermittent fasting, particularly TRE. Currently, evidence comparing TRE with standard daily caloric restriction (DCR) in terms of hepatic fat reduction in individuals with MASLD remains limited. In a randomized controlled trial involving adults with obesity and MASLD, TRE was associated with a ~ 6% reduction in intrahepatic triglyceride content, an average weight loss of approximately 7 kg, and improvements in metabolic parameters after 12 months. These results indicate that TRE may represent a feasible and effective dietary approach for selected patients, highlighting the importance of tailoring nutritional interventions to individual preferences and tolerability (43, 45).

Beyond dietary interventions, lifestyle modification in MASLD also relies heavily on structured physical activity. According to current recommendations, physical activity in individuals with MASLD should be personalized. It is generally advised to engage in more than 150 min of moderate-intensity aerobic exercise or at least 75 min of vigorous-intensity activity per week, combined with efforts to minimize sedentary behavior, to optimize both metabolic and hepatic outcomes (7). In particular, engagement in non-occupational physical activity has been shown to reduce the prevalence of MASLD and lower all-cause mortality (46). Regular, structured exercise improves insulin sensitivity, promotes weight loss, and reduces hepatic fat content (47). Both aerobic and resistance training modalities have shown beneficial effects in individuals with MASLD (48). However, compared with their well-established cardiometabolic benefits, the evidence supporting the impact of physical activity on histological improvement, non-invasive fibrosis markers, or liver-related clinical outcomes remains limited and inconclusive (7).

Finally, smoking cessation is strongly encouraged, as tobacco use negatively impacts disease progression and increases the risk of HCC and cardiovascular events (7, 49). Similarly, alcohol consumption should be minimized, and complete abstinence is advised for individuals with significant fibrosis (≥F2) or cirrhosis. Alcohol intake has been shown to promote fibrosis progression in a dose-dependent manner and to synergize with cardiometabolic risk factors, further aggravating hepatic injury (7, 50, 51).

#### Pharmacologic therapies for MASH

Pharmacological approaches for MASH are currently evolving, with agents targeting distinct pathophysiological mechanisms (52). Among non–glucose-lowering therapies, resmetirom, a liver-directed thyroid hormone receptor-*β* agonist, is the first FDA-approved drug for non-cirrhotic MASH with fibrosis (F2–F3), demonstrating histological improvements in steatohepatitis and fibrosis (28, 53). Other investigational agents include the FXR agonist obeticholic acid, which was shown to improve fibrosis but was not approved due to safety concerns (54), and lanifibranor, a pan-peroxisome proliferator-activated receptor (PPAR) agonist currently under phase III evaluation (55). Additionally, Vitamin E has shown histological benefit in non-diabetic MASH, while ursodeoxycholic acid and its derivatives, despite biochemical improvements, have failed to consistently demonstrate histological efficacy (56–59). Similarly, statins are considered safe and may reduce liver-related outcomes in MASLD, but evidence from randomized control trials confirming histological benefit is currently lacking (60). Lastly, n-3 PUFAs (EPA and DHA) possess anti-inflammatory and insulin-sensitizing properties yet have not consistently demonstrated histological benefit in clinical studies (61, 62). Notably, ongoing trials are evaluating modified formulations such as icosabutate—a structurally engineered fatty acid that has shown potential to suppress liver inflammation and fibrosis in preclinical models of MASH (63).

Glucose-lowering drugs, such as GLP-1 receptor agonists (e.g., liraglutide, semaglutide, tirzepatide) show promising results in steatohepatitis resolution through weight loss and metabolic modulation, though effects on fibrosis are limited (64). To enhance the therapeutic efficacy, dual and triple incretin co-agonists, targeting combinations such as GLP-1/GIP or GLP-1/GIP/glucagon, are currently under investigation (65, 66). By contrast, SGLT2 inhibitors such as empagliflozin and dapagliflozin have demonstrated modest reductions in hepatic fat and ALT levels, with no histological efficacy demonstrated to date from randomized trials (67).

Other drugs that have been used for the management of MASH include PPAR agonists, such as pioglitazone, a PPARγ agonist, which improve histological features of steatohepatitis (68, 69), yet their clinical use is limited by adverse effects and regulatory withdrawal in certain countries (70). Metformin does not appear to improve liver histology but may provide survival benefits in patients with advanced fibrosis or cirrhosis (71, 72). Overall, while resmetirom represents a landmark in MASH pharmacotherapy, further research is warranted to optimize monotherapy approaches, identify patient subgroups most likely to respond, and evaluate the potential of rational combination therapies for sustained disease modification.

### Management of MASH-HCC

Currently, there is no approved therapy specifically targeting both MASH-associatedHCC (73). The management of MASH-HCC involves treating the liver cancer according to the same oncologic guidelines applied to HCC of other etiologies, based on tumor stage, including resection, ablation, transplantation, transarterial chemoembolization (TACE), or systemic therapies such as atezolizumab, bevacizumab or tyrosine kinase inhibitors (74, 75). Notably, patients with MASH-related HCC may exhibit significant side effects from the chemotherapy, as well as reduced responsiveness to immunotherapy, possibly due to an altered immune microenvironment (6, 73, 76). In light of this, attention has turned to therapies that could target both the metabolic dysfunction and tumor progression. Agents such as polyunsaturated fatty acids (PUFAs) and NLRP3 inhibitors are being investigated for their dual antifibrotic and antitumor potential (77, 78). So far, they remain the only agents specifically investigated for dual action in MASH-HCC.

## Biochemistry and physiological role of PUFAs

Polyunsaturated fatty acids (PUFAs) are essential lipids whose physiological roles, despite extensive investigation, remain complex and at times contradictory. They are classified into omega-3 (n-3) and omega-6 (n-6) families derived from the essential precursors *α*-linolenic acid (ALA) and linoleic acid (LA), respectively (79). Although long-chain derivatives such as eicosapentaenoic acid (EPA; C20:5), docosahexaenoic acid (DHA; C22:6) and arachidonic acid (AA; C20:4) are widely believed to regulate inflammation and cardiometabolic health, the conversion of ALA to EPA/DHA and of LA to AA is inefficient, challenging assumptions about the functional adequacy of precursor-based intake (80). Therefore, the main dietary sources of the long-chain n-3 PUFAs EPA and DHA should be obtained from oily fish, whereas the long-chain n-6 PUFA AA should be predominantly obtained from animal-based products, particularly meat and poultry (81).

It is widely acknowledged that modern Western diets are characterized by a substantial insufficiency of n-3 PUFAs, typically exhibiting an n-6/n-3 ratio of 15–20:1 which far exceeds the proposed optimal ratio of approximately 4:1 and the ideal 1:1 ratio (82). However, achieving a more favorable n-6/n-3 balance does not necessitate restricting n-6 PUFA intake. Evidence indicates that higher n-6 PUFA consumption does not elicit adverse effects when accompanied by sufficient n-3 intake (83). Moreover, sufficient dietary provision of both n-3 and n-6 PUFAs is required to support optimal metabolic health and cardiovascular risk reduction (84). Beyond general metabolic health, PUFAs have been increasingly implicated in the prevention and management of MASLD and its progression toward HCC (85).

## Role of PUFAs in MASLD and MASLD-HCC prevention and management

### Role of PUFAs in the prevention of MASLD in humans

Human observational and genetic studies suggest that both n-3 and n-6 PUFAs may play a protective role in MASLD development (86, 87). In a large population-based analysis, higher circulating levels of total PUFAs and n-6 PUFAs, including linoleic acid, were independently associated with lower MASLD risk, while saturated fatty acids showed strong positive associations with disease prevalence (86). Mendelian randomization further supported a potentially causal inverse relationship between genetically predicted total PUFA levels, along with n-6 proportions, and MASLD risk (OR: 0.73 and 0.80, respectively) (86). n-3 PUFAs also show preventive potential. Large-scale prospective data from the UK Biobank indicate that regular long-chain n-3 supplementation (DHA) reduces the risk of liver disease, including MASLD, by approximately 28% (88) (Table 1). Although these studies provide evidence that PUFA insufficiency manifested by lower n-3 and n-6 levels may contribute to MASLD susceptibility, their observational nature necessitates caution as causality cannot be definitively established.

**Table 1:** Role of PUFAs in the prevention of MASLD and MASLD-HCC in humans.

Study	Country	Study design	Sample size	PUFAs investigated	Outcome measure	Major finding	Effect size
Liu et al., 2025 (86)	UK	Cross-sectional study	3,084	Omega-6 (linoleic acid)	Risk of incident MASLD	Circulating levels were negatively associated with MASLD risk	OR = 0.46 (95% CI: 0.27–0.79); *p*-value < 0.05
Liu et al.,2024 (88)	UK	Observational study	252,398	Omega-3 (DHA) and Omega-6	Risk of incident of MASLD-HCC	Plasma levels were negatively associated with MASLD-HCC risk	HR = 0.48 (95% CI: 0.33–0.69) and HR = 0.48 (95% CI: 0.28–0.81); *p*-value < 0.05 respectively
Moussa et al.,2021 (91)	USA	Case–control study	1,675	Omega-3 (EPA, DHA)	Risk of incident of MASLD-HCC	Intake was inversely associated with MASLD-HCC risk	OR = 0.50 (95% CI: 0.33–0.70); *p*-value < 0.05
Yang et al.,2020 (90)	USA	Prospective cohort study	138,483	Omega-3	Risk of incident of MASLD-HCC	Intake was inversely associated with MASLD-HCC risk	HR = 0.63 (95% CI: 0.41–0.96); *p*-value < 0.05

OR = odds ratio; HR = hazard ratio.

### Role of PUFAs in the prevention of MASLD-HCC in humans

Large-scale human data indicate a strong inverse association between circulating PUFA levels and the risk of progression from MASLD to HCC (89). In the UK Biobank cohort, elevated plasma concentrations of n-3 (DHA) and n-6 polyunsaturated fatty acids were associated with a substantially decreased risk of incident HCC and reduced mortality from chronic liver disease (88). Participants in the highest quartile of plasma n-3 and n-6 PUFA concentrations exhibited approximately a 50% reduction in HCC risk compared with those in the lowest quartile (88). Moreover, population-based studies similarly suggest that low PUFA intake may predispose individuals to MASLD-related HCC (90, 91). An analysis of data from two large U. S. prospective cohort studies indicates that higher dietary intake of PUFAs, particularly the n-3 subclass, is associated with a reduced risk of HCC (90). These findings are supported by results from a hospital-based case–control study, which similarly reported inverse associations between HCC risk and the intake long-chain n-3 PUFAs (EPA and DHA) (91). However, the evidence remains limited by heterogeneous populations and uncertainty regarding the influence of fibrosis stage on the association between PUFAs and HCC (88). Moreover, in a single-arm pilot study evaluating hepatic responses to n-3 supplementation in patients with MASLD and MASH, n-3 PUFA supplementation did not affect hepatic gene expression or histological features associated with HCC, indicating potentially limited effect of PUFAs in the prevention of HCC (Table 1) (92).

### Role of PUFAs in the management of MASLD in humans

Human interventional evidence evaluating PUFAs for MASLD management shows clinically meaningful metabolic improvements but inconsistent effects on liver histology (93). Meta-analyses of randomized controlled trials have reported reductions in liver enzyme (i.e., ALT, AST, *γ*-GT) levels, triglycerides, total cholesterol and liver fat following n-3 PUFA supplementation in MASLD (mainly EPA & DHA, some studies also included DPA) (Table 2) (94–96). However, heterogeneity across trials, particularly variations in dose, EPA/DHA composition and study duration has led to inconsistencies with some RCTs failing to show histological benefit, such as a change in fibrosis (61, 62). Therefore, although n-3 PUFAs have been consistently shown to improve metabolic parameters and reduce hepatic steatosis, their therapeutic efficacy in more advanced stages of disease remains unclear (97). Robust, long-term clinical trials are needed to define optimal dosing strategies and to establish clinically meaningful endpoints.

**Table 2:** Role of PUFAs in the management of MASLD and MASLD-HCC in humans.

Study	Country	Study design	No. of patients	PUFAs investigated	Daily dose (g)	Therapy duration	Major findings	Effect size
Kim et al., 2025 (97)	South Korea	Meta-analysis of 20 randomized controlled trials	1,615	Omega-3	1.5–4.0	3–12 months	Significant effect on gamma-glutamyl transferase (γGT), but not on AST, ALT, hepatic fat, stiffness or histology	γGT levels in MASLD: WMD = − 5.38 IU/L (95% CI: −9.16 – −1.61); *p*-value<0.05
Lee et al., 2020 (95)	Taiwan	Meta-analysis of 22 randomized controlled trials	1,366	Omega-3 (mainly EPA & DHA, some studies also included DPA)	1.0–4.0	3–12 months	Significant effect on triglyceride, total cholesterol and high-density lipoprotein levels in MASLD	Triglyceride levels: MD = − 28.57 (95% CI: −40.1 – −16.33) Total cholesterol levels: MD = − 7.82 (95% CI: −14.86 – −0.79) High-density lipoprotein levels: MD = 3.55 (95% CI: 1.38–5.73); *p* < 0.05
Argo et al., 2015 (62)	USA	Double-blind randomized, placebo-controlled trial	41	Omega-3 (EPA & DHA)	3.0	12 months	No significant effect for the primary endpoint of NAS reduction ≥ 2 points without fibrosis progression in MASLD	Four of 17 omega-3 (24%) and 3 of 17 placebo-treated patients (18%) had reduction in NAS (*p* = 0.99)
Sanyal et al., 2014 (61)	USA	Double-blind randomized placebo-controlled trial	243	Omega-3 (EPA)	2.7	12 months	Significant effect on triglyceride levels in MASLD, but not on steatosis, inflammation, fibrosis and liver enzymes	Levels of triglycerides: −6.5 mg/dL vs. +12 mg/dL in the placebo group (*p* = 0.03)

AST = aspartate aminotransferase; ALT = alanine aminotransferase; WMD = weighted mean difference; MD = mean difference; NAS = non-alcoholic fatty liver disease activity score.

### Role of PUFAs in the management of MASLD-HCC in humans

Although observational studies demonstrate marked alterations in PUFA status among affected patients, findings that may hold therapeutic relevance, the evidence supporting the clinical efficacy of PUFA supplementation in individuals with established MASLD-HCC remains extremely limited (88, 98). Collectively, evidence suggests that while circulating PUFA levels may serve as prognostic biomarkers for adverse hepatic outcomes, there is currently no evidence that PUFA supplementation can alter disease progression once MASLD has progressed to advanced fibrosis or HCC (Table 2) (88). Robust, targeted randomized controlled trials conducted specifically in MASLD-HCC populations are necessary to generate high-quality evidence before any safe conclusions about therapeutic effectiveness can be made.

### Role of PUFAs in MASLD and MASLD-HCC in cell and animal studies

Studies in mouse models and cell lines have also shown MASLD and HCC suppressive properties of PUFAs. Most studies in mouse models have shown the impact of PUFAs in MASLD. These studies involve feeding mice with variations of a high fat diet that induces MASLD and then adding interventions involving PUFAs. These interventions range from adding DHA alone, DHA and EPA, palmitoleic acid n-3 PUFAs, Deuterium-reinforced PUFAs, fish oil, *ω*-3 algal oil (rich in DHA) and ω-7 sea buckthorn oil (rich in palmitoleic acid), hemp seed oil, krill oil, soybean oil, seed oil of *rosa roxburghii* tratt, canola oil and unsaturated alginate oligosaccharides (99–117) (Table 3). Even though these studies assess slightly different outcomes related to MASLD, such as plasma lipids, hepatic fat, steatosis and fibrosis, all studies have shown that addition of these different PUFA interventions in the diets of mice results in suppression of MASLD progression. Some studies have shown that hepatic PUFA levels are reduced during MASLD progression, further attesting to their disease preventive role (118, 119) (Table 3).

**Table 3:** Role of PUFAs in MASLD and MASLD-HCC in animal studies.

Reference	Mouse model	PUFA intervention	Outcomes/results
Antraco et al. (99)	High fat diet (HFD)	Fish oil	↓ body/liver mass, plasma lipids/transaminases, glucose, cholesterol liver content
Liu et al. (100)	HFD	Fish oil	↓ hepatic steatosis
Hirako et al. (101)	High cholesterol	Fish oil	↓ Hepatic fat
Soni et al. (102)	HFD	EPA and DHA	↓ Hepatic triglyceride content, lipid/fatty acid biosynthesis
Hao et al. (103)	HFD	n-3 PUFAs	↓ body weight and fat mass
Wang et al. (104)	HFD	n-3 PUFAs	↓ MASLD
Smid et al. (105)	MCD diet*	n-3 PUFAs	↓ MASLD
Li et al. (106)	MCD diet	D-PUFAs**	↓ MASH
Wang et al. (107)	HFD	Palmitoleic acid	↓ liver injury, hepatitis, and dyslipidemia
Chen et al. (108)	HFD	DHA	↓ MASLD
Zhou et al. (109)	HFD	DHA	↓ MASLD
Nakamoto and Tokuyama (110)	CD^, 0.1% methionine-HFD	DHA	↓ Inflammation, fibrosis
Li et al. (111)	HFD	ω-3 algal oil (DHA rich) and *ω*-7 sea buckthorn oil (palmitoleic acid rich)	↓ lipid profiles, hepatic steatosis
Gong et al. (112)	MCD diet	Hemp seed oil	↓ hepatic steatosis, inflammation, fibrosis
Hwang et al. (113)	HFD	Krill Oil	↓ hepatic steatosis
Sanchez et al. (114)	HFD	soybean oil	↓ early MASH, glucose intolerance
Manca et al. (115)	HFD	Canola oil	↓ hepatic and retroperitoneal fat
Ni et al. (116)	HFD	seed oil of *Rosa roxburghii* Tratt	↓ MASLD progression, lipid accumulation, oxidative stress, inflammatory response
Cha et al. (117)	Growth hormone receptor knockout	Unsaturated alginate oligosaccharides	↓ insulin resistance, hepatic steatosis (lean MASLD)
Ishii et al. (122)	Pten deficient	EPA	↓severe chronic hepatic inflammation, ROS formation, HCC development
Inoue-Yamauchi et al. (123)	HFD + carcinogen	EPA	↓HCC development
Yan et al. (118)	HFD	N/A	↓ n-3 FAs, n-3/n-6 in mice fed HFD
Xavier et al. (119)	CD L-amino-defined diet	N/A	↓ PUFAs in MASLD
Vlock et al. (120)	CD HFD	N/A	↓ PUFAs in lean MASH-HCC
Hymel et al. (121)	CD and CS¶ HFD	N/A	↓ PUFAs in lean and obese MASH-HCC

* Methionine, choline deficient, ** Deuterium-reinforced polyunsaturated fatty acids, ^CD = choline-deficient, ¶CS = choline supplemented.

Only a handful of studies in mice have addressed the role of PUFAs in MASLD-HCC progression. In a study of a non-obese MASLD-HCC mouse model, reduced plasma and tumor tissue levels of DHA and EPA were reported, as well an increase of these PUFAs over time in mice that never progress to HCC, implying their potential tumor suppressive role (120). Furthermore, a comparison of obese and non-obese MASLD-HCC mice showed a reduction of PUFAs in both models during tumor progression (121) (Table 3). A different study of Pten deficient mice, showed that EPA supplementation resulted in reduction of severe chronic hepatic inflammation, ROS formation, and HCC development (122). In another model of HCC induced by high fat diet consumption and carcinogen exposure, EPA supplementation resulted in reduction of HCC development (123) (Table 3). Finally, some work regarding the role of PUFAs in HCC was carried out in cell lines derived from HCC of viral etiologies. EPA and DHA treatment resulted in inhibition of HCC cell growth in three different cell lines through inhibition of COX-2 and beta-catenin, thereby providing some evidence of the role of PUFAs in HCC (124).

## Mechanisms mediating the effects of PUFAs on MASLD pathogenesis

**Figure 3:**
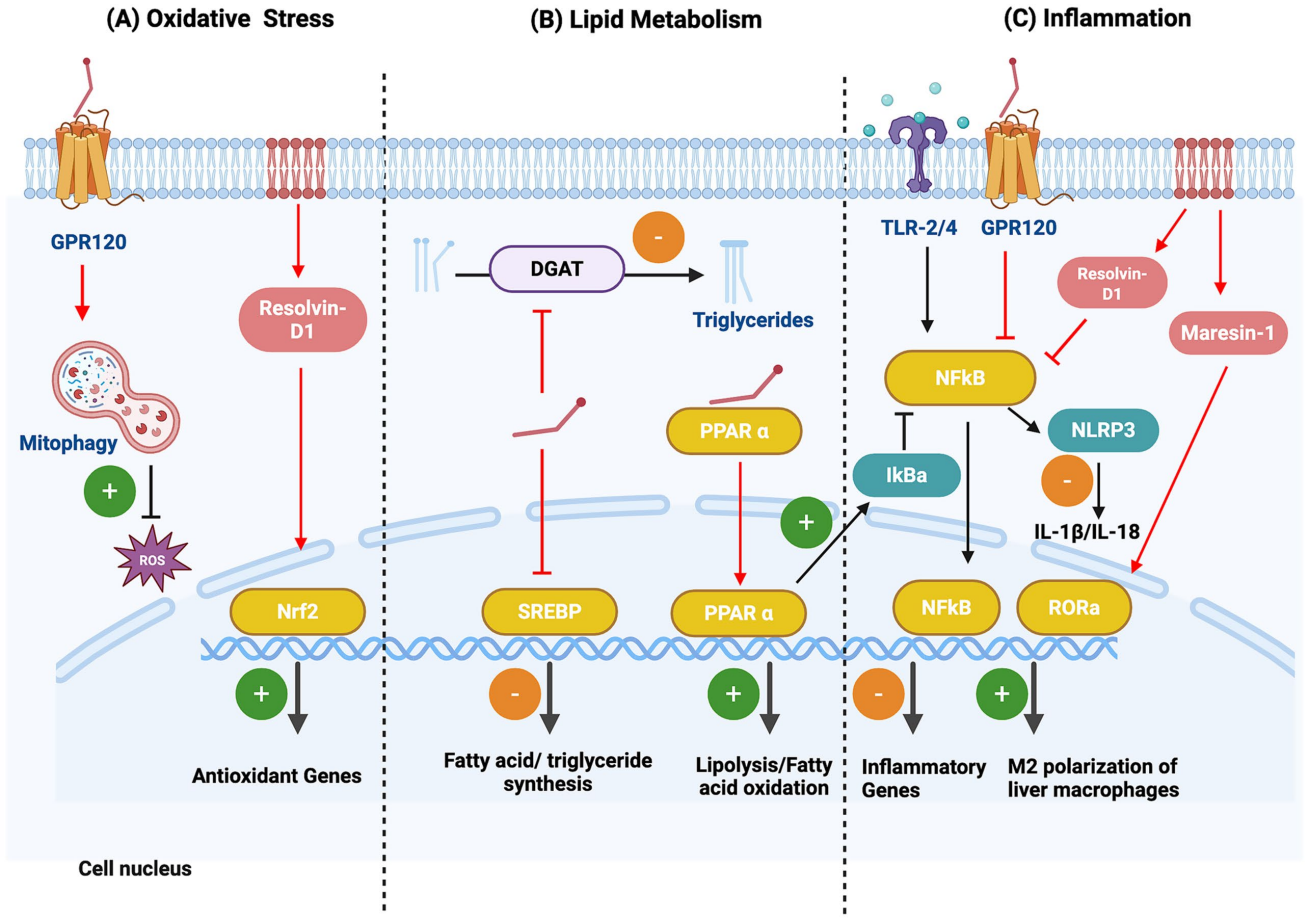
Molecular mechanisms mediating the effects of n3-PUFAs on MASLD. n-3-PUFAs have a protective role against the development of MASLD by exerting antioxidant (A) and anti-inflammatory effects (C), and by regulating hepatic lipid metabolism (B). (A) n-3 PUFAs promote mitophagy of damaged mitochondria, by activating the GPR120 receptor, leading to a reduction in ROS levels. The DHA-derived mediator Resolvin-D1 promotes the expression of antioxidant genes by activating the cytoprotective transcription factor, Nrf2. (B) n-3 PUFAs induce lipolysis and fatty acid oxidation by activating the nuclear receptor PPARα while at the same time reducing fatty acid and triglyceride synthesis through inhibition of the transcription factor, SREBP. Furthermore, n-3 PUFAs reduce triglyceride synthesis by inhibiting the enzyme DGAT. (C) The anti-inflammatory effects of n-3 PUFAs are mediated through inhibition of NFκB. Specifically, activation of GPR120 receptor and the DHA-derived mediator Resolvin D1 lead to inhibition of TLR-mediated NFκB activation. Also, PPARα activation promotes the expression of the NFκB inhibitor, IκBα. Inhibition of NFκB by n-3 PUFAs also leads to reduced NLRP3 inflammasome activity. Finally, the DHA-derived Maresin-1 promotes M2 polarization of liver macrophages, preventing the early development of MASH, by activation of the nuclear receptor, RORα. Created in https://BioRender.com.

**Figure 4:**
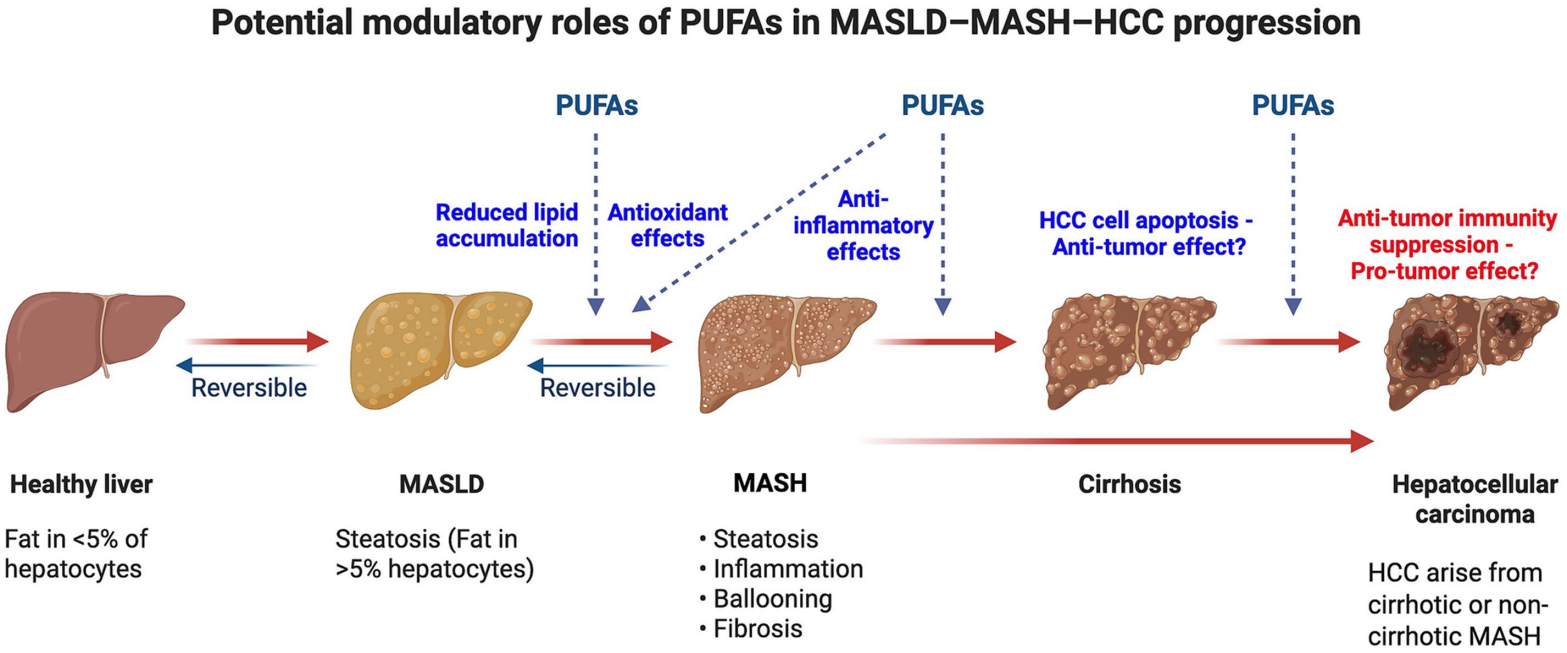
The effects of n3-PUFAs on MASLD progression to MASH and HCC. Proposed modulatory effects of polyunsaturated fatty acids (PUFAs) on metabolic dysfunction, inflammatory processes, and potential antitumor effects in HCC across the MASLD–MASH–HCC continuum. Created in https://BioRender.com.

Several studies have demonstrated a protective effect of n-3 PUFAs in metabolic diseases including MASLD through their effects on lipid metabolism, inflammation and oxidative stress (Figures 3, 4). Indeed, n-3 PUFAs were shown to reduce markers of metabolic dysfunction, oxidative stress, and liver inflammation and injury in a LDLR-deficient western diet-induced mouse model of MASLD (125).

### Effect of PUFAs on metabolic dysregulation in MASLD

Metabolic impairment and hepatic lipid accumulation are central to the pathogenesis of MASLD. n-3 PUFAs have been shown to reduce hepatic steatosis by driving a shift from lipogenesis to lipid catabolism in hepatocytes. More specifically, *in vitro* studies have demonstrated that n-3 PUFAs inhibit the activity of sterol regulatory element-binding protein 1 (SREBP1), which is a transcription factor that regulates genes involved in fatty acid and triglyceride synthesis. This effect is mediated by reducing SREBP1 expression, through inhibition of gene transcription and induction of mRNA degradation, and by preventing the proteolytic release of SREBP1 from the endoplasmic reticulum (126–128). At the same time, n-3 PUFAs are ligands of the nuclear receptors PPAR, which drive lipolysis and fatty acid oxidation, and inhibit lipogenesis (129, 130). n-3 PUFAs are also known to inhibit the activity of diacylglycerol acyltransferase (DGAT), an enzyme catalyzing the terminal step of hepatic triglyceride synthesis, in the liver (131). These findings are confirmed in clinical and experimental studies of MASLD. A study in obese MASLD patients showed an increased n-6 PUFA/n-3 PUFA ratio, which was associated with increased SREBP1 and decreased PPARα expression, suggesting increased lipogenesis (132). DHA and olive oil supplementation were also shown to increase PPARα expression and transcriptional activity, whilst reducing SREBP1 expression and activity, in a high fat diet-induced mouse model of MASLD (133). Moreover, in a high-fat diet mouse model, EPA reduced hepatic triglyceride levels and altered the composition of VLDL by promoting increased unsaturated fatty acid content. The latter is mediated by inducing the expression of the enzyme stearoyl-CoA desaturase-1 (SCD-1), which catalyzes the conversion of saturated to unsaturated fatty acids (134).

### Effect of PUFAs on oxidative stress responses in MASLD

Lipid accumulation in the liver leads to impaired mitochondrial function and subsequently increased production of ROS. This leads to the development of oxidative stress, resulting in hepatic injury and the release of pro-inflammatory DAMPs. n-3 PUFAs are known to prevent oxidative stress, thus potentially mitigating hepatic damage, and consequently inflammation and fibrosis (Figures 3, 4). EPA and DHA were shown in *in vitro* studies to act as direct antioxidants by scavenging ROS, whilst they can also activate the cytoprotective transcription factor nuclear factor erythroid 2–related factor 2 (Nrf2) (135, 136). Indeed, J_3_-isoprostanes, products of non-enzymatic n-3 PUFA oxidation were shown to activate Nrf2 by preventing its degradation by the Keap1-Cullin3 E3 ubiquitin ligase complex in hepatocytes (137). Furthermore, in an MCD mouse model of MASH, the DHA-derived mediator Resolvin D1 was reported to induce Nrf2-mediated antioxidant responses and prevent oxidative damage (138). DHA was also shown to protect hepatocytes from oxidative stress-induced injury by promoting the removal of dysfunctional mitochondria through mitophagy, mediated by GPR120, a G protein–coupled long-chain fatty acid receptor (139).

### Effect of PUFAs on inflammatory pathways in MASLD

DAMPs released due to hepatic injury, as well as bacterial endotoxins arising from gut dysbiosis, promote hepatic inflammation and fibrosis through activation of TLRs, particularly TLR-2 and TLR-4, and downstream signaling pathways, including MAPKs, NFκB and the NLRP3 inflammasome (140–143). n-3 PUFAs have been shown to play a key role in protection against hepatic inflammation in MASH (142) (Figures 3, 4). EPA and DHA were reported to inhibit TLR-2 and -4 activity, by reducing their expression and the systemic abundance of their agonists, in high-fat diet LDLR^−/−^ mouse models of MASH. This effect was associated with a reduction in NFκB activity and hepatic inflammation and fibrosis (125, 144). In a high fat diet mouse model, activation of GPR120 by n-3 PUFAs was shown to inhibit TLR-mediated signaling in macrophages by inhibition of downstream kinases, and through receptor internalization via its adaptor protein *β*-arrestin2 (145). n-3 PUFAs also inhibit high fat diet-induced activation of NFκB, possibly by increasing the expression of its inhibitor IκB*α* in a PPARα-dependent manner (146, 147). Resolvin D1, an endogenous lipid mediator derived from DHA through a lipoxygenase-mediated mechanism, also inhibited TLR-4-induced NFκB and MAPK activation in an MCD mouse model of MASH (138). Maresin-1, another DHA-derived pro-resolving mediator, was shown to activate the nuclear receptor retinoic acid receptor-related orphan receptor (ROR)α, which drives macrophage M2 polarization inhibiting liver inflammation and preventing early MASH development (148). n-3 PUFAs also inhibit the activation of the NLRP3 inflammasome, a key multiprotein complex that drives the caspase 1-mediated cleavage, maturation, and secretion of the proinflammatory cytokines IL-1β and IL-18. Activation of NLRP3 depends on two signals; a priming signal that involves TLR/NF-κB-dependent expression of NLRP3 and pro–IL-1β, and an activation signal triggered by cellular stressors, including ROS, that induces NLRP3 complex assembly and caspase-1 activation (149). n-3 PUFA supplementation was shown to prevent NLRP3 priming, through NFκB inhibition, in a high fat diet-induced mouse model of MASH (150).

## Mechanisms mediating the effects of PUFAs on HCC development in MASLD

There are a limited number of experimental studies investigating the effect of PUFAs, specifically n-3 PUFAs, on MASLD-HCC development. These studies have shown that the effects of n-3 PUFAs may be context-dependent and can be either protective or detrimental.

### Protective effects of PUFAs against MASLD-HCC development

In an alcohol/high-fat/high-sugar mouse model of MASLD, the patatin-like phospholipase domain-containing protein 3 (PNPLA3) variant I148M, which is associated with increased n-3 PUFAs levels, conferred protection against HCC development (151). Furthermore, a diet-induced lean MASH-HCC mouse model, mice that developed HCC showed lower plasma n-3 and n-6 PUFA levels. The reduction in PUFA levels, possibly caused by a reduction in desaturase expression, was associated with tumor progression (120). These findings indicate a role of n-3 PUFAs in preventing the development of HCC in MASLD patients.

The mechanisms underlying this protective effect are poorly understood. EPA supplementation led to reduced hepatic steatosis and inflammation and prevented the development of HCC in a PTEN-deficient mouse model of MASH. Moreover, EPA reduced the proliferation of primary hepatocytes isolated from the mouse model, by inhibiting extracellular signal-related kinase (ERK)1/2 MAPK activity (122). These results suggest that the anti-tumorigenic effect of n-3 PUFAs may be mediated through inhibition of pro-inflammatory pathways. In a different high-fat MASH mouse model, EPA was shown to supress the activation of the oncogenic transcription factor STAT3, and prevent diethylnitrosamine-induced HCC development, without affecting hepatic inflammation (123). Furthermore, *in vitro* studies have reported that EPA and DHA promote apoptosis and inhibit the proliferation of HCC cell lines through different mechanisms, including inhibition of the Wnt/*β*-catenin and COX-2 activity and activation of c-Jun N-terminal protein kinase (JNK) and p53 signaling pathways (124, 152–156). Nano-liposomes containing 2,6-di-isopropylphenol-linolenic acid conjugate were also shown to induce apoptosis and inhibit the migration and adhesion of the HCC line HepG2 (157). Whether this would apply to MASLD-HCC-derived cell lines remains to be determined.

### Detrimental effects of PUFAs on MASLD-HCC development

A recent study demonstrated a negative effect of PUFAs on anti-tumor immunity. More specifically, mucosal-associated invariant T (MAIT) cells, which exert liver anti-tumor immunity by inducing HCC cytotoxicity, show accumulation of n-3 and n-6 PUFAs in MASLD patients. The same study demonstrated that arachidonic acid and DHA, promote ROS-dependent impairment of mitochondrial respiration and glycolysis, leading to metabolic exhaustion of MAIT cells. In addition, exaggerated PUFA-mediated lipid peroxidation was shown to trigger MAIT cell ferroptosis. This leads to a reduction in the numbers and bioenergetic capacity of MAIT cells, negatively affecting their tumour-killing ability (158).

The effect of n-3 PUFAs, on HCC development in MASLD patients may therefore be cell type and stage-specific, which introduces another layer of complexity to potential therapeutic applications of PUFAs.

## Conclusions/discussion

There is increasing evidence through observational studies involving large cohorts that PUFAs play a role in the prevention of MASLD and MASLD-HCC. In addition, randomized control trials have shown a therapeutic potential of PUFAs in MASLD, however, the evidence remains poor regarding the role of PUFAs in MASLD-HCC treatment. Animal models have also shown extensively that different combinations of PUFA supplementation results in reduction of MASLD outcomes, further supporting the role of PUFAs in MASLD prevention and management. Evidence for the role of PUFAs in MASLD-HCC prevention stems from observations that PUFA levels are reduced during MASLD-HCC progression and different mouse models showing reduction of HCC development with PUFA supplementation. The mechanistic basis of the role of PUFAs in MASLD and MASLD-HCC lies on their effects in lipid metabolism, inflammation and oxidative stress. Therefore, PUFAs help with reducing fat accumulation, reducing inflammation and alleviating oxidative stress, which contributes to MASLD and MASLD-HCC prevention. On the other hand, understanding the effect of PUFAs in HCC treatment remains elusive and controversial with one study showing that PUFAs may actually have a suppressive role of anti-tumor immune response. This controversial effect may potentially stem from differing effects of PUFAs depending on tumor stage and characteristics, such as tumor microenvironment, which can be rich in ECM (fibrosis/cirrhosis), exhibit inflammation, and/or recruitment of various types of immune cells.

In summary, the evidence for n-3 PUFAs as a preventive agent in early MASLD and its progression to HCC is compelling. Their role in altering the course of established HCC, however, remains a mystery. The role of PUFAs in HCC appears to be context-dependent and may be a double-edged sword, potentially suppressing tumours in some contexts while impairing anti-tumour immunity in others. This paradox represents the key frontier for future research.

### Future directions

While there is a lot of evidence supporting the role of PUFAs on MASLD and MASLD-HCC prevention and management, there are some gaps that need to be filled by further research. At the clinical level, design of RCTs specifically in patients with advanced fibrosis (F3-F4), the group at highest risk for HCC, are needed to clarify if PUFA supplementation can delay or prevent carcinogenesis. Such clinical studies can be further supplemented by mechanistic studies using MASLD-HCC-specific models to untangle the cell-type-specific effects of PUFAs and clarify whether PUFAs have a protective effect on hepatocytes and detrimental effect on immune cells. Tools such as single-cell sequencing and metabolomic analyses on PUFA-treated models can provide answers to these questions and potentially contribute to the development of guidelines for context-dependent use of PUFAs in clinical practice based on tumor immune microenvironment status. Furthermore, translational studies can be designed to address the role of PUFAs in combination with existing cancer therapies for MASLD-HCC, such as immunotherapy and tyrosine kinase inhibitors.
